# Functionalization of Cellulose Nanocrystals in Choline Lactate Ionic Liquid

**DOI:** 10.3390/ma9070499

**Published:** 2016-06-23

**Authors:** Sarah Montes, Itxaso Azcune, Germán Cabañero, Hans-Jürgen Grande, Ibon Odriozola, Jalel Labidi

**Affiliations:** 1 Materials Division, IK4-CIDETEC Research Centre, Paseo de Miramón 196, 20009 San Sebastián, Spain; iazcune@cidetec.es (I.A.); gcabanero@cidetec.es (G.C.); hgrande@cidetec.es (H.-J.G.); iodriozola@cidetec.es (I.O.); 2 Chemical and Environmental Engineering Department, University of the Basque Country, Plaza Europa 1, 20018 San Sebastian, Spain; jalel.labidi@ehu.eus

**Keywords:** cellulose nanocrystals, functionalization, ionic liquid, poly(lactic acid) (PLA), nanocomposite

## Abstract

Cellulose nanocrystals (CNCs) are valuable nanomaterials obtained from renewable resources. Their properties make them suitable for a wide range of applications, including polymer reinforcement. However, due to their highly hydrophilic character, it is necessary to modify their surface with non-polar functional groups before their incorporation into a hydrophobic polymer matrix. In this work, cellulose nanocrystals were modified using a silane coupling agent and choline lactate, an ionic liquid derived from renewable resources, as a reaction medium. Modified cellulose nanocrystals were characterized by infrared spectroscopy, showing new peaks associated to the modification performed. X-ray diffraction was used to analyze the crystalline structure of functionalized cellulose nanocrystals and to optimize the amount of silane for functionalization. Poly(lactic acid) (PLA) nanocomposites containing 1 wt % of functionalized cellulose nanocrystals were prepared. They were characterized by field-emission scanning electron microscopy (FE-SEM) and mechanical tests. The use of choline lactate as reaction media has been shown to be an alternative method for the dispersion and silanization of the cellulose nanocrystals without the addition of an external catalyst.

## 1. Introduction

Cellulose, which is considered as the most abundant biopolymer on earth [1], is a promising feedstock from lignocellulosic biomass due to its availability and renewability. Cellulose nanocrystals (CNCs) are stiff rod-shaped particles with nanoscale dimensions. They consist of pure crystalline cellulose chain segments, which are usually isolated from cellulosic fibers through an acid treatment to remove amorphous domains [2]. These nanomaterials exhibit high specific strength and modulus, high surface area, and low density (~1.6 g·cm^-3^). These distinctive properties together with their non-toxic character make them suitable materials for a wide range of applications, such as biosensing, green catalysis, synthesis of drug carriers in therapeutic and diagnostic medicine [3,4], carrier materials for catalysts [5,6,7], stimuli responsive molecules [8,9,10], nanopaper fabrication [11], and preparation of polymer nanocomposites [12].

In the field of polymer nanocomposites, CNCs have been successfully used as reinforcement agents for both synthetic and natural polymers [13,14,15,16]. The increasing interest in new environmentally friendly plastics has promoted the development of fully biobased materials. Thus, polymers like poly(lactic acid) (PLA) have been used in the preparation of CNC-reinforced nanocomposites for improving thermal and mechanical properties [17,18,19].

Homogeneous dispersion of CNCs within the polymer matrix and good matrix-filler interaction are key factors in achieving an excellent performance of nanocomposite properties. Due to the hydrophilic character of CNCs, the incorporation into water-soluble matrices is the simplest method for using CNCs as filler [20]. Water is a very convenient processing medium for composite materials, because the high stability of aqueous CNCs dispersions endows their good dispersion within the host matrix. However, when it comes to nonpolar polymeric matrices, the high hydrophilic character of CNCs prevents a good dispersion. Consequently, chemical modification of CNC surfaces is of great interest in order to improve their compatibility with a wider variety of polymer matrices [21].

CNCs have a very high surface-to-volume ratio with plenty of hydroxyl groups on their surface that can be used for surface functionalization via a wide range of chemical approaches. Thus, the compatibility with the matrix can be enhanced by introducing a suitable functionality. Commonly used surface functionalizations for CNCs are esterification, etherification, oxidation, amidation, carbamation, nucleophilic substitution, silanization, polymer grafting, etc. [22].

Hydrophobitation of cellulose is an interesting strategy for enhancing the compatibility with non-polar polymer matrices. Recently, stearyl groups have been successfully reported for this purpose [23]. Similarly, silanization has proven to be an effective method of providing hydrophobicity [24]. Silanes are known as efficient coupling agents for improving the interfacial adhesion between the reinforcement and matrix and have been extensively used in composites [25,26]. However, there are several aspects that need to be addressed. On the one hand, this type of reaction is usually performed in organic polar solvents. The use of organic solvents entails several drawbacks related to their potential toxicity, emission of VOCs, and the generation of large waste byproducts causing environmental problems. On the other hand, the silanization reaction of CNCs is usually promoted by basic or acid catalysts, as they accelerate the hydrolysis of alkoxy groups to yield silanol groups able to react with –OH groups present on CNC surfaces. Nevertheless, this approach simultaneously promotes the condensation reaction between adjacent silane molecules that yield to the formation of silica, instead of the functionalization of surface. Thus, a simple and environmentally friendly method of performing an efficient silanization of CNCs is still desirable.

In the last decade, ionic liquids (ILs) have emerged as effective and environmental friendly alternatives to volatile organic solvents, mainly due to their almost negligible vapor pressure and their tunable physical and chemical properties. ILs are liquid organic salts usually consisting of an organic cation and a polyatomic anion that can be selected from a wide chemical pool. As a matter of fact, ILs have been used in the replacement of volatile organic solvents in a wide variety of chemical processes, such as the separation, purification, and reaction media in biochemical and chemical catalysis [27,28,29]. ILs have been successfully used in biomass pretreatment to be further hydrolyzed into glucose sugars [30]. It is worth noting that cellulose is poorly soluble in conventional solvents, mainly because of the large amount of intermolecular hydrogen bonds present in its structure. Interestingly, ILs are capable of disrupting such hydrogen bonds between different microfibrils while decreasing the compactness of cellulose and making the carbohydrates more susceptible to hydrolysis [31,32,33,34,35,36]. ILs have also been used in the preparation of cellulosic materials [37,38,39] and as reaction media in the processing of cellulose derivatives [40,41,42]. 

The aim of this study was to improve CNC compatibility with poly(lactic acid) by their surface functionalization. The functionality was introduced onto the CNCs by using 2-(carbomethoxy)ethyltrimethoxysilane (2CETMS) as a silane coupling agent and choline lactate IL, derived from renewable sources, as a reaction medium.

The effect of this IL on the functionalization of CNCs was investigated, and several silane concentrations were studied. Interestingly, we have experimentally observed that, along with its capacity of properly dispersing CNCs without dissolving them, choline lactate does not require the addition of any external catalyst to promote the reaction, in contrast to other reaction media used for the same reaction [24].

The functionalized CNCs (F-CNCs) were subsequently applied as reinforcements in a PLA matrix. Good quality PLA nanocomposites films were prepared by solution casting followed by hot pressing. Dispersibility of F-CNCs in PLA and mechanical properties of nanocomposites were also investigated.

## 2. Results and Discussion

### 2.1. Preparation of CNCs

**Figure 1:**
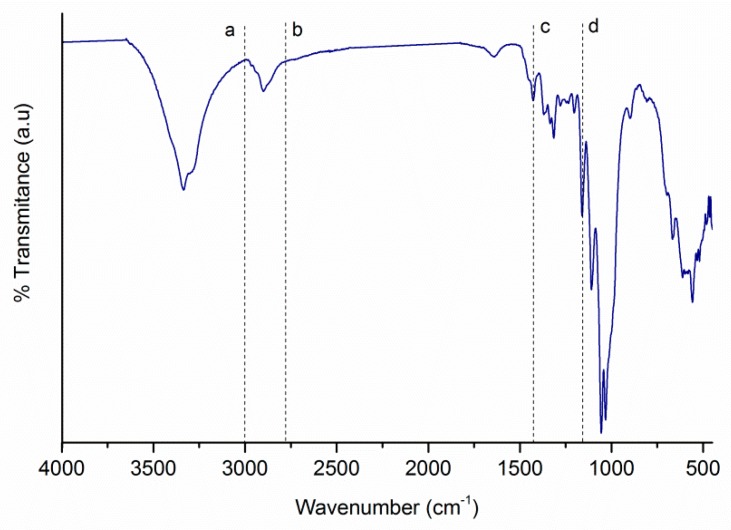
FTIR spectrum of CNCs obtained from acid-hydrolysis of MCC.

CNCs were obtained by acid-hydrolysis of microcrystalline cellulose (MCC) using a procedure described elsewhere [43]. CNCs were analyzed with Fourier transform infrared spectroscopy (FTIR) and field-emission scanning electron microscopy (FE-SEM). Figure 1 shows the FTIR spectrum of the starting CNCs. Bands between 3600 and 3000 cm^−1^ corresponding to the –OH stretching vibration were observed. The band between 3000 and 2750 cm^−1^ was assigned to asymmetric and symmetric C–H stretching vibration (a,b). The peaks observed at ~1430 cm^−1^ were assigned to CH_2_ symmetric bending (c), and the ones at 1160 cm^−1^ were assigned to asymmetric C–O–C bridge stretching (d), and are associated to the crystalline part of the cellulose.

**Figure 2:**
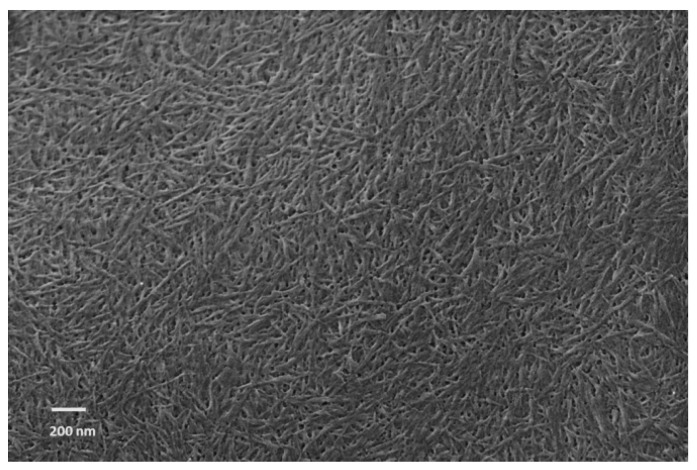
FE-SEM micrograph of a freeze-dried specimen of CNCs.

Figure 2 shows a FE-SEM micrograph of agglomerated CNCs. The lengths of CNCs were in the range from 200 to 250 nm, and the average diameter was calculated to be 18 ± 4 nm.

### 2.2. Functionalization of CNCs

Two types of CNCs were used to perform the functionalization reaction, sulfated and desulfated. Sulfated CNCs were directly obtained after acid-hydrolysis with H_2_SO_4_ and subsequent dialysis, whereas, in the case of desulfated CNCS, surface sulfate groups were hydrolytically cleaved by alkaline hydrolysis in order to increase the number of available surface hydroxyl groups. Success of the desulfation reaction was confirmed via elemental analysis (Table 1). Sulfated CNCs show a sulfur content of 0.53%, while, in desulfated CNCs, this content is in the lower limit of detection of the apparatus.

**Scheme 1:**
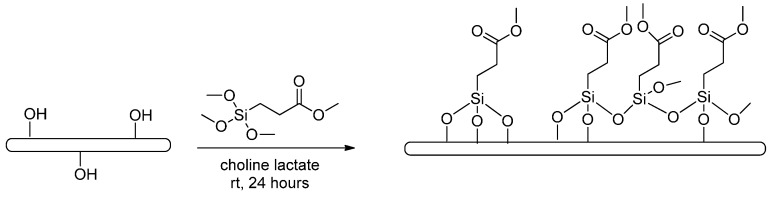
Proposed scheme reaction for CNCs functionalization.

Then, both CNCs, sulfated and desulfated, were functionalized using choline lactate IL as solvent and CETMS as silane coupling agent, as shown in Scheme 1.

The CNC: Silane ratio was varied to optimize the amount of silane needed to carry out the reaction. Other reaction conditions, such as the time of reaction and the presence of an additional solvent were also studied. The results are summarized in Table 2.

**Figure 3:**
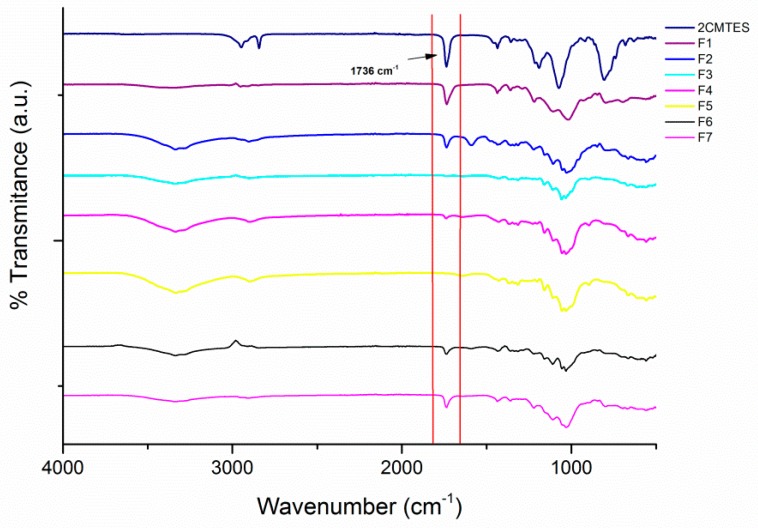
FTIR spectra of 2-(carbomethoxy)ethyltrimethoxysilane (2CMETS) and functionalized CNCs (F-CNCs) prepared in different conditions.

FTIR and X-ray diffraction analysis (XRD) techniques were used to evaluate the functionalization of CNCs. Figure 3 shows the spectra of all F-CNCs (from F1 to F7) obtained in different reaction conditions. The spectrum of 2-(carbomethoxy)ethyltrimethoxysilane (2CMETS) is also shown for comparative purposes. In the spectra of silanized CNCs, a new peak can be observed at ~1736 cm^−1^ corresponding to the carbonyl group of the 2CMETS fragment. This evidence of functionalization was found for F1, F2, F4, F6, and F7, but it was not present for F3 and F5, indicating that silanization had not taken place. In the case of F3, the reaction time was not enough, whereas, in F5, the addition of choline lactate to ethanol, used as a reaction medium, did not lead to CNC functionalization. Nevertheless, an advanced structure study would be required to fully characterize the structural binding of silane onto CNCs, which is beyond the scope of the present paper. Additionally, thermogravimetric analysis (TGA) was used in order to quantify the amount of residual solids after the reaction (mainly SiO_2_). This residue is derived from the amount of grafted silane but also from the condensation reaction between adjacent silane molecules or even part of the reagent that might have been adsorbed on the surface. The largest amount of solids was found for F1, which reached 45%.

**Figure 4:**
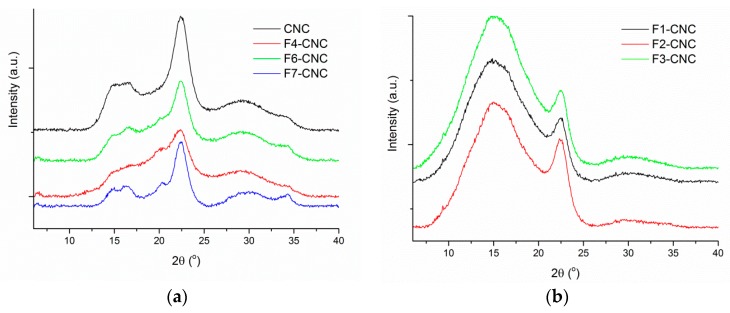
Wide angle X-ray scattering (WAXS) of F-CNCs under optimized reaction conditions (**a**) and under excess of silane (**b**).

The structures of F-CNCs were characterized by wide angle X-ray scattering (WAXS) in order to study the effect of the reaction conditions on the crystallinity of CNCs. Figure 4a shows WAXS curves for naked CNCs and F4, F6, and F7 functionalized CNCs. All of them exhibited three main reflection peaks at 2θ = 15.0°, 16.3°, and 22.5°, as well as a fourth, less defined one at 34.4°, relative to the cellulose I crystalline structure. In Figure 4b, CNCs diffractograms obtained under F1, F2, and F3 conditions are compared. These conditions correspond to high silane ratios in the reaction medium and therefore lead to higher amounts of amorphous SiO_2_ as reaction byproduct from the condensation reaction. The WAXS curves for the nanocrystal peaks at 2θ = 15.0° and 16.3° were not identified at these conditions, but a broad shoulder at around 2θ = 16° was found instead, typical of a predominantly amorphous material.

As proof of concept, F7 conditions were selected to prepare PLA nanocomposities. This selection was based on the following: (i) the starting CNCs were sulfated (desulfation step could be skipped); (ii) CNCs were used from the aqueous dispersion (better dispersion than re-dispersing from freeze dried CNCs); and (iii) the molar ratio AGU:silane was the lowest tried that did not affect to the crystallinity, according to the WAXS experiments.

### 2.3. Preparation of PLA Nanocomposites

**Figure 5:**
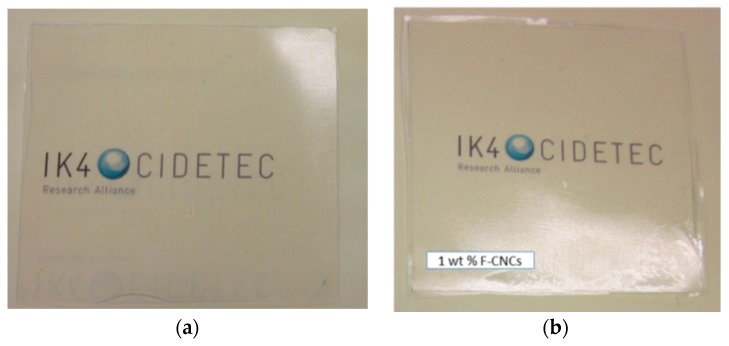
Poly (lactic acid) (PLA) nanocomposite films prepared by hot pressing: (**a**) neat PLA and (**b**) PLA with 1 wt % of F-CNCs.

To evaluate if the silanization of CNCs was a feasible strategy for improving the compatibility towards PLA, films containing 1 wt % of F-CNCs (obtained under F7 reaction conditions) were prepared. For that aim, PLA was dissolved in THF, and predispersed F-CNCs in THF were added to the PLA solution. The mixture was then placed in a Petri dish until a solvent evaporation occurred. For the production of a transparent film, the so-obtained PLA/CNC nanocomposite was processed at 180 °C in a hot press for 15 min. For comparative purposes, neat PLA films were also prepared. As is shown in Figure 5, the quality of PLA films was very good and no evidence of CNCs agglomeration was observed with the naked eye.

#### 2.3.1. Morphological Characterization

**Figure 6:**
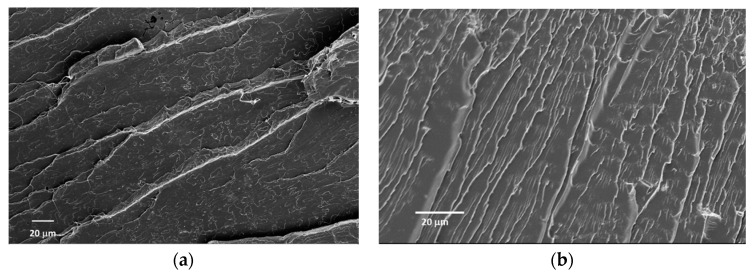
FE-SEM micrographs of cryogenically fractured surfaces of (**a**) neat PLA and (**b**) 1 wt % of F-CNC/PLA nanocomposites.

Figure 6 shows the FE-SEM micrographs of the cryogenic fractured surfaces of neat PLA and PLA-based nanocomposite containing 1 wt % of F-CNCs. Neat PLA showed a surface typical of a semicrystalline polymer in accordance to previously reported literature [44]. The fractured surface corresponding to PLA 1 wt % of F-CNCs appeared to be flatter and have plenty of fracture lines, suggesting a more brittle material than neat PLA [45]. The lack of evidence regarding the formation of agglomerates may be interpreted as a sign of good dispersion.

#### 2.3.2. Mechanical Characterization

**Figure 7:**
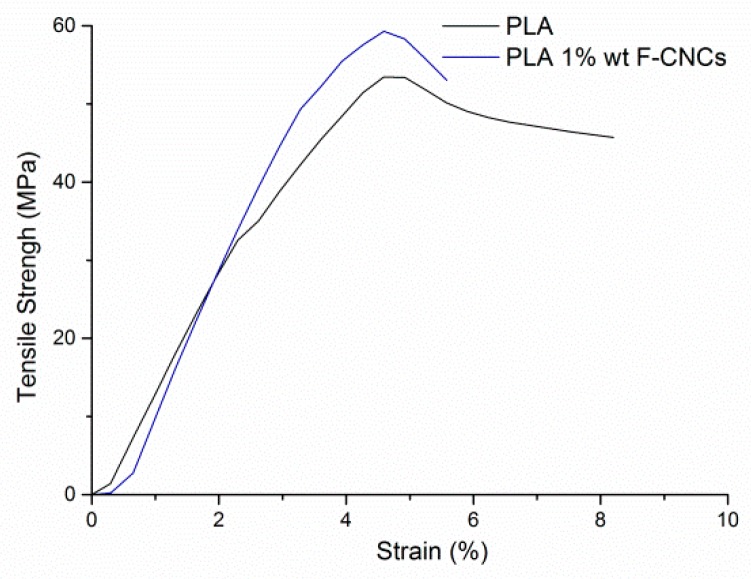
Representative stress-strain curves for neat PLA and 1% wt F-CNC reinforced PLA nanocomposite.

Figure 7 shows the stress–strain curves of neat PLA and PLA with 1 wt % of F-CNCs, while the main representative mechanical parameters are summarized in Table 3.

Neat PLA presented a semi-ductile behavior with a strain at a break of 8.2%. The lowering of elongation at break with the addition of reinforcements is a common trend observed in thermoplastic polymer composites. As was expected, the addition of a 1 wt % of F-CNCs increased tensile strength at expenses of ductility.

The addition of 1 wt % of F-CNCs improved Young’s modulus (E) and the tensile strength (σ) in 2% and 4%, respectively, indicating that the modification of CNCs improved their dispersion degree and that the interfacial adhesion between PLA and CNCs was enhanced.

## 3. Materials and Methods

### 3.1. Materials

2-(carbomethoxy)ethyltrimethoxysilane (2CMETS) was purchased from Fluorochem (Derbyshire, UK), PLA pellets (Ingeo™ 3052D, Minnetonka, MN, USA) were purchased from Nature Works LLC, and microcrystalline cellulose (MCC) and tetrahydrofurane (THF) were purchased from Sigma-Aldrich (Madrid, Spain). Sulfuric acid (H_2_SO_4_, 98%) and sodium hydroxide (NaOH) were purchased from Scharlau (Barcelona, Spain).

Choline chloride (99% purity) was obtained from Algry Química (Huelva, Spain). Lactic acid (Galacid™, Scannafles, Belgium, Heat Stable 90, 86%) was obtained from Galactic. AMBERLITE^®^ IRA400 Cl, sodium hydroxide ACS reagent grade (97%), and deuterated DMSO (dimethyl sulfoxide-d6, 99.5 %D) were purchased from Sigma-Aldrich.

CNCs were extracted from microcrystalline cellulose (MCC) by acidic hydrolysis with sulfuric acid according to a protocol reported elsewhere [43]. Isolation protocol is described in Section 3.3.

### 3.2. Synthesis of Choline Lactate

Choline lactate was synthesized by anion-exchange chromatography technique. A 0.1 M solution of choline chloride (1.0 equiv., 100 mmol, 14.38 g) was passed through a anion-exchange Anberlite resin column charged with hydroxide anion and was collected over lactic acid (1.0 equiv., 100 mmol, 10.41 g) solution. Water was evaporated via rotary evaporator and the product was obtained as a pale oil (yield = 19.3 g, quantitative). ^1^H NMR (500 MHz, DMSO-*d*_6_) 3.86–3.83 (m, 2H, HOCH_2_CH_2_N^+^(CH_3_)_3_), 3.63 (q, 1H, *J* = 6.7 Hz, HOCHCH_3_CO_2_^−^), 3.41 (t, 2H, *J* = 5.0 Hz, HOCH_2_CH_2_N^+^(CH3)_3_), 3.12 (s, 9H, HOCH_2_CH_2_N^+^(CH_3_)_3_), 1.11 (d, 3H, *J* = 6.7 Hz, HOCHCH_3_CO_2_^−^).

### 3.3. Functionalization of CNCs

Prior to silanization, CNCs obtained after acidic hydrolysis were pre-treated in order to investigate the effect of such pre-treatments on the subsequent functionalization. Thus, CNCs were used in three different forms: sulfated, in aqueous dispersion and desulfated.

#### 3.3.1. Functionalization of Sulfated CNCs

Sulfated CNCs were used as obtained after acidic hydrolysis. Briefly, MCC powder (20 g) was added to a preheated solution of 64% *w*/*w* sulfuric acid (175 mL) and reacted with vigorous stirring at 45 °C for 45 min. To quench the reaction, the reaction mixture was diluted in 5 L of deionized water and allowed to settle overnight. In order to concentrate the CNCs and remove the excess of acid and water, the suspension was centrifuged at 3500 rpm for 30 min. The precipitate was repeatedly rinsed and centrifuged with deionized water until the supernatant became turbid. Further, CNC purification was done by dialysis against deionized water until reaching a pH of 5–6 (membrane Spectra/Por 2, MWCO 12,000–14,000). Finally, the CNCs were freeze-dried.

For functionalization, 300 mg of sulfated CNCs were dispersed in 16.4 g of choline lactate and stirred for 15 min at room temperature. Then, 249 mg of 2CETMS silane coupling agent were added while stirring. The reaction was kept 24 h at room temperature. Then, the functionalized CNCs were rinsed by centrifugation for 30 min at 3500 rpm with deionized water and freeze-dried until used.

#### 3.3.2. CNCs in Aqueous Dispersion

The CNCs were prepared following the previous protocol until the dialysis process. The obtained CNC aqueous dispersion was neutralized with NaOH until pH ~ 7. For functionalization, CNCs were dispersed in 16.4 g of choline lactate and freeze-dried to remove water. After that, 249 mg of the 2CETMS silane coupling agent were added, and the reaction was kept 24 h at room temperature, and the same protocol as that in Section 3.3.1 for rinsing and storing were followed.

#### 3.3.3. Desulfated CNCs

Desulfated CNCs were prepared following the protocol reported by Lokanathan et al. [46]. Briefly, concentrated NaOH (aq) was added to an 8.9% *w*/*w* suspension of CNC until a concentration of 7% *w*/*v* NaOH was obtained. After 30 min. of stirring at room temperature, the mixture was stirred for 5 h. at 65 °C. After 5 h, the reaction mixture was rinsed with water to remove the excess of NaOH and dialyzed (membrane Spectra/Por 2, MWCO 12,000–14,000) against deionized water. Finally, the CNCs were freeze-dried. For functionalization the same protocol as that described in Section 3.3.1 was followed, varying the ratio of CNC:silane, the time of reaction, and the incorporation of a mixture of IL and ethanol as the reaction medium (see Table 2). The effectiveness of desulfatation was determined by elemental analysis [47].

### 3.4. Preparation of F-CNCs/PLA Nanocomposites

For the preparation of F-CNCs/PLA nanocomposites, 20 g of PLA were dissolved in 300 mL of THF at 60 °C. Then, 0.5 wt % and 1 wt % of F-CNCs previously dispersed in THF were added to the polymer solution and stirred for 20 min. The blends were evaporated at room temperature and dried under vacuum for 5 h at 60 °C.

For film formation, the blends were hot pressed in a hot plate press at 180 °C and 200 bar for 15 min. For comparative purposes, neat PLA films were prepared under the same conditions.

### 3.5. Characterization Techniques

Fourier transform infrared (FTIR) spectroscopy was recorded at room temperature on a Nicolet Avatar 360 spectrophotometer (Madison, WI, USA). The spectra were taken with a 4 cm^−1^ resolution in a wavenumber range from 4000 to 400 cm^−1^.

Elemental analysis was performed using a LECO Micro Truspec CHN (St. Joseph, MI, USA) to measure sulfur, carbon, nitrogen, and hydrogen contents independently.

Thermogravimetric analysis (TGA) was performed in a TGA Q500 (Thermal Analysis Instruments, New Castle, DE, USA).

The X-ray diffraction pattern was recorded by using a Bruker AXS-D8 Advance X-ray diffractometer (XRD, Madison, WI, USA) with CuKa radiation. The measurement was carried out between 2θ values from 5° to 40°.

The tensile properties of the nanocomposites (Young’s Modulus, tensile strength, and elongation at break) were evaluated using a tensile test with a universal testing machine model 3365 (Instron, Grove City, PA, USA). The initial length of the test specimens (12 mm × 65 mm, with a thickness between 300 and 350 microns) was 25.4 mm and a cross head speed of 50 mm∙min^−1^ was used. The number of tested specimens for the mechanical properties was 10 for average calculations.

Scanning electron microscopy was carried out with a Carl Zeiss Ultra Plus field-emission scanning electron microscope (FE-SEM, Oberkochen, Germany) equipped with an energy dispersive X-ray spectrometer (EDXS). NMR spectra were recorded in a Bruker Avance III NMR spectrometer (Billerica, MA, USA) using deuterated DMSO.

## 4. Conclusions

In the present work, the hydrophilic character of cellulose nanocrystals was modified by a silanization process. This process was carried out using choline lactate, an IL derived from renewable resources, as a reaction medium. The polarity of the IL and the moderate basic character of the lactate anion might presumably favor the solvation of the CNC surface and the hydrolysis of methoxysilane group. FTIR results confirmed that the chemical modification of CNCs was successfully achieved in some of the studied conditions. Additionally, the crystallinity of the CNCs, determined by XRD, was similar after chemical modification, except for the excess of silane conditions, which led to the condensation of SiO_2_.

Nanocomposites, using PLA reinforced with 1 wt % functionalized CNCs, were prepared and characterized. Morphological characterization showed a lack of agglomerates. Additionally, the tensile tests showed an enhancement in mechanical properties (Young’s modulus and tensile strength). These results showed that the modification with silane could contribute to the improvement in interfacial bonding between the filler and matrix.

Furthermore, the use of choline lactate IL has demonstrated to be an effective reaction medium for performing the silanization of CNCs without modifying their crystalline structure.

## Figures and Tables

**Table 1:** % S by weight (expressed as elemental sulfur) for sulfated and desulfated CNCs.

Sample	% S
Sulfated CNCs	0.53 ± 0.03
Desulfated CNCs	0.04 ± 0.0001

**Table 2:** Reaction conditions of functionalization of CNCs in choline lactate ionic liquid.

F-CNCs	CNC Pretreatment	CNC:Silane Molar Ratio	IL:CNC Ratio *w*/*w*	Reaction Time (h)	TGA Residue (%)
F1	Desulfated	1:10	55:1	24	45.48
F2	Desulfated	1:1.25	55:1	24	27.13
F3	Desulfated	1:0.6	55:1	4.5	No Functionalization
F4	Desulfated	1:0.6	55:1	24	11.68
F5	Desulfated	1:0.6	0.81:8.1 Ethanol	24	No Functionalization
F6	Sulfated	1:0.6	55:1	24	20.13
F7	Aqueous dispersion	1:0.6	55:1	24	12.09

**Table 3:** Mechanical behavior of PLA-based nanocomposite films.

Material	E Modulus (MPa)	σ (MPa)	ε at Break (%)
PLA	1780 ± 48	52.5 ± 4.2	7. 28 ± 0.41
PLA/1 wt % F-CNCs	1815 ± 176	54.7 ± 5.1	5.30 ± 0.31
